# #sleepingbaby on Instagram: Nonadherence of images to safe sleeping advice and implications for prevention of Sudden Unexpected Death in Infancy

**DOI:** 10.1371/journal.pone.0290580

**Published:** 2023-09-13

**Authors:** Floortje Kanits, Monique P. L’Hoir, Magda M. Boere-Boonekamp, Adèle C. Engelberts, Edith J. M. Feskens

**Affiliations:** 1 Department of Global Nutrition, Division of Human Nutrition and Health, Wageningen University and Research, Wageningen, the Netherlands; 2 Department of Health Technology & Service Research, University of Twente, Enschede, the Netherlands; 3 Department of Pediatrics, Zuyderland Medical Center, Sittard-Geleen, the Netherlands; Noble Shivapuri Research Institute, NEPAL

## Abstract

**Objectives:**

Safe sleep of infants is important to reduce the risk of Sudden Unexpected Death in Infancy (SUDI). The depiction of infant care behavior which is inconsistent with the safe sleep recommendations on social media has an impact on parental infant care thoughts, norms and behaviors. This study aims to determine the adherence of Instagram images to the Dutch safe sleeping advice.

**Design:**

A systematic social media analysis on Instagram was performed using 22 hashtags and 9 accounts of Dutch companies or platforms related to infants. Images of sleeping infants were analyzed on consistency with the criteria: supine sleeping position, own cot or crib, sleep sack, and an empty bed.

**Results:**

Based on 514 collected images, 5.9% was consistent with sleep sack use, 16.8% with an empty bed, 30.7% with an own cot or crib, and 67.5% with the supine sleeping position. For 311 images (60.5%), all four criteria could be rated, as for the others, at least one criterion was not clearly depicted. Only 6 of these images (1.9%) were consistent with all four criteria.

**Conclusions:**

Although Instagram images are probably not representative of regular infant care behavior, the exposure to these images that are mostly inconsistent with the safe sleep advice can contribute to the formation of norms, and therefore influence parental care behavior. Accurate communication of the safe sleep recommendations through social media is needed, and opportunities are described for preventive health professionals to engage more in this communication with their public.

## Introduction

Safe infant sleep is important in the prevention of Sudden Unexpected Death in Infancy (SUDI). SUDI is a broad term used to describe the sudden unexpected death of an infant without an apparent cause, which includes Sudden Infant Death Syndrome (SIDS). SIDS is “the sudden unexpected death of an apparently healthy infant under one year of age that remains unexplained after a thorough case investigation, including performance of a complete autopsy with ancillary testing, examination of the death scene, and review of the clinical history,” and often occurs during an unobserved sleep period [1, 2]. In the Netherlands, 27 infants (0.15 per 1000 live births) died suddenly and unexpectedly in 2021, according to national statistics [3].

The risk of SUDI increases when a vulnerable infant in a critical developmental period is exposed to an exogenous stressor(s) [4]. Risk factors for SUDI can be unavoidable (e.g. an infection), or avoidable (e.g. the prone sleeping position). Avoidable risk factors relate to infant care practices of the parents or caregivers.

To minimize the risk of SUDI, prevention advice aims to influence this parental infant care behavior. The most important safe sleeping recommendations include always put the infant to sleep in the supine position; in its own infant bed or cot, in the parents’ bedroom; without a duvet, pillow, or other soft (bedding) material; and in a sleep sack (‘Four of Safe Sleeping’, VeiligheidNL, n.d.). Furthermore, parents are advised not to smoke prenatally and postnatally, to breastfeed the infant if possible, and to consistently place the infant to sleep with a pacifier [5, 6].

Attitudes and behaviors are also shaped by other factors, such as content shared over social media. According to the Theory of Normative Social Behavior, descriptive norms (people’s perceptions about the prevalence of a behavior) affect individuals’ own behaviors through interactions with three normative mechanisms: injunctive norms, outcome expectations, and group identity [7]. Injunctive norms reflect individual’s perceptions of what behaviors are expected of them by important others, and social sanctions will be suffered if they do not comply. Outcome expectations are the likely consequences of engaging in a particular behavior. Group identity encompasses an individual’s aspiration to become more like peers and one’s perceived similarity to their peers. Social media can be an important source of information about descriptive and injunctive norms, and contribute to the formation of group identities [8].

Since the beginning of the twenty-first century, social media claims an increasingly important place in daily life. The number of social media channels used daily, and the time that is spent on them is increasing. On average in the Netherlands, 4.4 platforms are used per person aged 15 years and older, of which 2.4 platforms on a daily basis, responsible for an average daily time commitment of 107 minutes per person [9, 10].

With approximately one billion active users per month, Instagram is one of the most popular social media platforms in the world. Users can share photos and videos, or look at and interact with others’ posts. More than two thirds of all Instagram users are under 35 years of age [11]. The daily use of Instagram is declining among teenagers, while growth is in the 25 and over age groups [9].

This target audience of Instagram also includes young or future parents. Therefore, the depiction of sleep-related infant care behaviors has implications for parental thoughts, norms, and behaviors about infant care. Parents of young children who are exposed to images on Instagram showing infant care behaviors that are inconsistent with the safe sleep advice may therefore adopt this risk-increasing behavior. This may lead to an increase in SUDI incidence. To reinforce SUDI prevention, it is important for healthcare providers to know what is happening on social media and the potential influence thereof on parental behavior. Therefore, this study aims to determine the adherence of Instagram images of apparently sleeping infants to the Dutch safe sleeping advice.

## Methods

A systematic social media analysis on Instagram was performed using hashtags relevant to infant sleeping practices, and accounts of Dutch companies or platforms related to infants. Images of infants with closed eyes (apparently sleeping) were included and analyzed on adherence to Dutch safe sleeping advice, defined as the four most important recommendations: ‘the 4 of Safe Sleeping’ (‘De 4 van Veilig Slapen’, [12]).

## Materials

First, twenty-two hashtags were used to search for a relevant sample of Instagram images of apparently sleeping infants. The hashtags used were determined by conducting an initial cursory search on Instagram; the hashtags that yielded the highest frequency of relevant searches, with a minimum of 1.000, were used (Table 1). Only general terms were included so that specific searches for consistent or inconsistent behavior with the safe sleep advice were not included.

**Table 1:** Used hashtags for the Instagram search, including an English translation, with the number of hits in May 2022.

Hashtags	*English translation*	Hits
#babybed	*baby bed*	237.805
#babybedje	*little baby bed*	3.800
#babyinspiratie	*baby inspiration*	6.464
#babykamer	*baby room / nursery*	309.230
#babykamertje	*little baby room / nursery*	1.744
#babyopkomst	*baby on the way*	104.894
#babyproduct	*baby product*	253.650
#babyproducten	*baby products*	11.419
#babyslaap	*baby sleep*	3.073
#babyslaapcoach	*baby sleep coach*	3.059
#babyslaapt	*baby is asleep*	1.874
#babyuitzet	*baby stuff / layette*	89.788
#babywieg	*baby cot*	1.899
#doorslapen	*sleep through*	8.382
#dutje	*nap*	10.721
#dutjedoen	*take a nap*	4.586
#ledikant	*crib*	13.560
#ledikantje	*little crib*	3.924
#slaapkindjeslaap	*sleep baby sleep*	7.080
#slaaplekker	*sleep well*	76.351
#slapendebaby	*sleeping baby*	1.644
#wieg	*cot*	11.585

For each hashtag, the 200 latest posts were collected with an Instagram hashtag scraper (https://github.com/arc298/instagram-scraper), with the following code: instagram−scraper−−tag {hashtag}−u {Instagram username}−p {Instagram password}−m 200−t image−d./{hashtag}. Only public images were scraped, with no other information such as account names. As a result, images could not be traced back to individuals. Because of Copyright protection, these data cannot be published publicly and permission is needed to access the data.

The images were screened on inclusion criteria: infant with closed eyes and estimated to be under the age of 1 year. Excluded were images of infants apparently in a hospital and/or an incubator. Duplicate images were deleted. Some images were very similar, for example the same infant in the same sleep environment, but the photo was taken from another angle. In these cases, only the image where the sleep environment was most clearly depicted was included for analysis.

Secondly, 10 large Instagram accounts of Dutch companies or platforms targeted at parents of young infants, with at least 10.000 followers (Table 2) were scraped similarly. The 200 latest posts per account were collected and screened on the same criteria.

**Table 2:** Used Dutch companies/platforms on Instagram with the number of followers in May 2022.

Dutch companies/platforms	Rounded number of followers
Prénatal (@prenatal_nl)	97.000
Oei ik groei (@oeiikgroei)	63.000
Babypark (@babyparknl)	41.000
24baby.nl (@24baby.nl)	37.000
Babydump (@babydump)	23.000
BabyPlanet (@babyplanetnl)	13.000
MiniMe.nl (@minimemagazine)	13.000
Pampers (@pampers_nl)	12.000
Ouders van nu (@oudersvannu)	78.000
Yumi Baby Holland (@yumibabyholland)	47.000

In total, 562 unique photos were collected. On the account of @babyparknl, no images fulfilling the inclusion criteria were found. Forty-eight photos were excluded because of high similarity with another included photo. In total, 514 photos were included for analysis, of which 381 were collected by hashtag searches, and 133 through accounts of companies/platforms. The proportion of images meeting the inclusion criteria was unknown prior to data collection and may vary significantly with a repetition of the data collection. Consequently, the study population size was dependent on the posted images rather than being pre-specified.

## Analysis

Included images were analyzed on adherence to the Dutch safe sleeping advice by rating the consistency (yes/no/uncertain) of the images with each item of ‘the 4 of safe sleeping’-recommendations (Table 3). Consistency per image was rated by two researchers (first author and research assistant) independently. A Cohen’s Kappa coefficient was calculated for each criterion to measure the inter-rater reliability. Subsequently, any discrepancies between the researchers were discussed to reach agreement.

**Table 3:** Analysis criteria on consistency with Dutch safe sleep recommendations.

The Four of Safe Sleeping (VeiligheidNL, n.d.)		Consistent with the Dutch recommendation (yes)	Inconsistent with Dutch recommendation (no)	Consistency not sure
When I put my infant to sleep,. . .	. . .I put it on its back.	Supine position	Side, prone position	Not clear enough to see.
	. . .I put it in its own cot or crib.	Crib, cot, bedside cosleeper / click-on bed, Moses basket, portable crib, bassinet	Bed (any size); sitting device; couch, sofa, chair; in-bed co-sleeper, positioner, sleep nest; play pen/yard; on parents’ lap/chest; carrier or pram	Not clear enough to see.
	. . .I put it in a sleep sack.	Well-fitting sleep sack; swaddled in accepted sacks^a^	No sleep sack, or sleep sack with armholes or neck opening that are too large; swaddled in cloths or non-recommended sacks^a^; there is no clear zipper or row of buttons visible on the front of the baby	Not clear enough to see.
	. . .I put it in an empty bed.	Only baby (with sleep sack, if applicable) possibly with tightly tucked bedding (sheet and/or blanket) on the sleep surface; pacifier and tucked in hydrophilic cloth are accepted	(Soft) toys; another person or animal; untucked blanket(s), pillow, bumper, sleep nest, or other soft bedding; duvet; cushioning of sides	Not clear enough to see.

^a^Pacco, Boelie Originals, Woombie, Ergococoon and Puckababy are the only types of swaddling-cloths or -sleep sacks described as safe when used properly by the Dutch Consumer Association (Consumentenbond) and are therefore accepted.

The consistency of images with each recommendation was compared between images found via hashtags and those of companies/platforms using the Pearson Chi-square statistic. A p-value below 0.05 was considered statistically significant.

## Results

The agreement between the two raters of the images was considered moderate to almost perfect, with κ being 0.55 for the criterion ‘own cot or crib’, 0.59 for ‘empty bed’, 0.79 for ‘sleep sack’, and 0.88 for ‘supine sleeping position’ (all p<0.001). A final agreement between the raters was reached for all existing discrepancies.

For 311 of the 514 (60.5%) images, all four criteria could be rated. For the other 203 images (39.5%), the consistency with at least one criterion was uncertain as it was not clearly depicted in the image. For the supine sleeping position, it was uncertain in 7 images (1.4%), for the own bed in 148 (28.8%), for the sleep sack in 76 (14.8%), and for the empty bed in 7 images (1.4%).

When clearly depicted, only 5.9% of the infants were found to be sleeping in a sleep sack; 16.8% in a bed without toys, persons, or loose bedding materials; 30.7% in an own cot or crib, and 67.5% in the supine position (Table 4). In total, six images were consistent with all four recommendations.

**Table 4:** Consistency of images with safe sleep recommendations.

	Total images (N = 514)		Images having all 4 criteria rated (n = 311)
	n (%)		n (%)
**Supine position**	n = 505	341 (67.5)	218 (70.1)
**Own cot/crib**	n = 365	112 (30.7)	93 (29.9)
**Sleep sack**	n = 438	26 (5.9)	23 (7.4)
**Empty bed**	n = 507	85 (16.8)	42 (13.5)
**Consistent with all four recommendations**			6 (1.9)

N indicates the number of images that could be rated with certainty on the respective recommendation.

In Table 5, consistency per criterion is described separately for images found via hashtag search (n = 381) and through companies or platforms (n = 133). On images collected via Instagram accounts of Dutch companies or platforms targeted at parents of young infants, less infants were sleeping in an own cot/crib (21.3%), in a sleep sack (0.8%) and in a completely empty bed (11.3%), compared to images collected via hashtags (p<0.05).

**Table 5:** Images found through hashtag search and companies or platforms and their consistency with safe sleep recommendations.

	Images via hashtag search (n = 381)		Images via companies/platforms (n = 133)		p-value
	n (%)		n (%)		
**Supine position**	n = 374	254 (67.9)	n = 131	87 (66.4)	0.7
**Own cot/crib**	n = 285	95 (33.3)	n = 80	17 (21.3)	0.04
**Sleep sack**	n = 319	25 (7.8)	n = 119	1 (0.8)	0.006
**Empty bed**	n = 374	70 (18.7)	n = 133	15 (11.3)	0.05
**Consistent with all four recommendations**	n = 242	5 (2.1)	n = 69	1 (1.4)	0.7

N indicates the number of images that could be rated with certainty on the respective recommendation. P-value of the Pearson Chi-square test.

## Discussion

This study showed a low adherence to the Dutch safe sleeping advice in 514 Instagram images of sleeping infants. The highest consistency was found for the supine sleeping position (66%), while the lowest was observed for sleep sack use (5%). In total, six out of 311 images were consistent with all four recommendations. According to the Theory of Normative Social Behavior, images on social media can contribute to the formation of descriptive and injunctive norms, and therefore influence behavior [7]. Parents of young children who are exposed to these images on Instagram that are mainly inconsistent with the safe sleep recommendations may therefore be influenced to normalize or accept SUDI risk-increasing infant care.

Images posted by companies and platforms had the lowest consistency with the safe sleeping advice. The larger number of followers on company or platform accounts, compared to ordinary accounts, could influence the normative mechanisms of the viewers’ behavior. An individual’s perception of what behaviors are expected of them (injunctive norms) are likely to be higher for behaviors shown by important accounts, or those with many followers. Also, aspiration to become more like others (group identity) might be influenced by the importance or the number of followers of an account. However, not many images per account could be included (average 13.7, range 0–51), suggesting not many sleeping infants were posted in general.

Images of infants are often meant to be cute and do not show the actual daily care behavior [13]. In a Dutch national survey on infant safe sleeping practices in 2017, 72% of the infants was sleeping in the supine position, 92% in an own cot or crib, 56% in a sleep sack, and 39% in a bed without soft materials such as toys and/or pillows [14]. This suggests a higher adherence to the recommendations in the Dutch population than depicted on the Instagram images in the current study. Therefore, Instagram images are probably not representative for usual parental infant care behavior. However, it is unknown how this representativeness is perceived by caregivers. When caregivers do perceive the depicted behavior as usual behavior, the influence on the formation of descriptive and normative norms might be larger than when it is not perceived as usual behavior.

A comparable study conducted in the United States [15] found that 117 out of 1563 Instagram images (7.5%) obtained through 27 English hashtag searches, were consistent with the AAP safe sleep guidelines. However, on 1134 images (73%) a sleeping infant was depicted, of which only 43% was sleeping supine. Chin et al. (2021) also reported an unknown sleeping location in almost 60% of the images with a sleeping infant. The consistency of images with the Dutch safe sleep recommendations in the current study was observed to be lower compared to the findings of the US study, confirming the importance of studying this issue in different cultures and countries. Besides providing data of Dutch images and recommendations, the current study also specifically focused on influential accounts of companies or platforms, where the consistency with the recommendations of posted images was even lower. In contrast to Chin et al. (2021), this study considered images to which individuals may be exposed when their Instagram algorithm is tailored to infants and infants’ products, potentially affecting parental care behavior as described in the Theory of Normative Social Behavior [7]. Not only on social media, but also stock photos, used for websites and advertisements, often do not adhere to the safe sleep guidelines [16]. Of 1590 stock photos of sleeping infants, 30 photographs (1.9%) depicted an infant with all the correct basic elements of safe sleep recommended by the AAP [16].

Besides the unconscious influence of images on the formation of norms around parental care, caregivers are also influenced by the information about infant care they get through actively searching websites and social media. In addition, information is provided to caregivers via the more traditional routes such as Child Health Care (CHC) centers, which are visited by over 90% of the Dutch population [17]. In a US study, about 50% of people reported using social media or the internet for information on how and where their baby should sleep [18]. The information is most often found on popular websites or on Facebook and Instagram. It is unknown if this information is always reliable and if caregivers find and use official information platforms. This reliability is probably difficult to rate for caregivers themselves.

## Recommendations

There seem to be three important sources of information for parents on safe sleep: first, the unconscious influence of their social environment; second, the conscious search for information, mostly online, and third, the information they receive at CHC centers. These sources also provide opportunities for the prevention of SUDI.

Regarding the unconscious influence, there are opportunities to prevent high exposure of images on social media that do not comply with the safe sleep recommendations. Especially (influential) companies, platforms, and individuals with many followers should be aware and held accountable for the possible consequences of posting images of unsafe-sleeping infants. However, these platforms can also be used to raise awareness and distribute the correct information, for example, by creating descriptive and injunctive norms around the recommended safe sleep behavior.

Besides images on Instagram, caregivers are also exposed to conflicting information and beliefs around safe sleep via internet and other social media platforms such as Twitter [19] and Facebook [20]. Herein lies an opportunity for CHC centers and other health care providers to address common misconceptions and beliefs around safe sleep, provide accurate information to parents, and talk about the reliability of information they find on the internet and social media.

In general, increasing knowledge of caregivers about the safe sleep recommendations and SUDI is needed. Although Instagram images of sleeping infants are unlikely to be representative for the usual infant care behavior, most infants in the images were in a SUDI-risk increasing position or environment. Communication of safe sleep recommendations through CHC centers is still a very important and valuable channel. However, it is also important to anticipate and provide more modern, picture-driven information to reach all young parents, for example through Instagram. Currently, there is a lack of presence of public health organizations on social media.

## Limitations

To make the analysis of Instagram images as objective as possible, strict criteria were used to rate the images. This meant that if an image depicted an infant in a crib with a small stuffed animal in the corner of the crib, the crib was considered not empty, and therefore the image was not consistent with the safe sleeping recommendation. It can be argued if such small items in the infants’ sleep environment form a substantial risk for SUDI. Furthermore, of the 305 images with all criteria rated and being inconsistent on at least one of them, about 25% (n = 78) showed an awake adult. Infant care behavior inconsistent with the safe sleep recommendations, but under supervision of an awake adult may not increase the risk of SUDI substantially. The approach used in this study could therefore be too conservative, resulting in an underestimation of the consistency with the safe sleep recommendations. Finally, it is important to acknowledge limitations regarding generalizability in our study. The sample of analyzed images was obtained exclusively from Instagram accounts in the Netherlands and with specific Dutch hashtags. Therefore, it may not fully represent the entirety of images related to infant sleep on the platform. Nevertheless, comparable results were found in a similar study in the United States. Furthermore, only publicly shared images were included in our analysis, which excludes content from private accounts and restricts the generalizability of our findings.

## Conclusions

The inconsistency of images on social media with the safe sleep recommendations, combined with conflicting information on the internet in general, emphasizes the need for accurate communication through images and text with infant care providers. Although images on social media are probably not representative for actual infant care behavior, the exposure to inconsistent images contributes to the formation of norms, and can therefore influence parental care behavior.
